# Remaining Useful Life Prediction of Rolling Bearings Based on Multi-Scale Attention Residual Network

**DOI:** 10.3390/e25050798

**Published:** 2023-05-14

**Authors:** Lin Song, Jun Wu, Liping Wang, Guo Chen, Yile Shi, Zhigui Liu

**Affiliations:** 1 School of Information Engineering, Southwest University of Science and Technology, Mianyang 621010, China; 2 School of Intelligent Manufacturing, Panzhihua University, Panzhihua 617000, China; 3 State Key Laboratory of Tribology, Institute of Manufacturing Engineering, Department of Mechanical Engineering, Tsinghua University, Beijing 100084, China; 4 Strategic Technology and Equipment Development Center, China Academy of Engineering Physics, Mianyang 621010, China

**Keywords:** remaining useful life, feature reuse multi-scale convolutional, attention mechanism, lightweight

## Abstract

The remaining useful life (RUL) prediction of rolling bearings based on vibration signals has attracted widespread attention. It is not satisfactory to adopt information theory (such as information entropy) to realize RUL prediction for complex vibration signals. Recent research has used more deep learning methods based on the automatic extraction of feature information to replace traditional methods (such as information theory or signal processing) to obtain higher prediction accuracy. Convolutional neural networks (CNNs) based on multi-scale information extraction have demonstrated promising effectiveness. However, the existing multi-scale methods significantly increase the number of model parameters and lack efficient learning mechanisms to distinguish the importance of different scale information. To deal with the issue, the authors of this paper developed a novel feature reuse multi-scale attention residual network (FRMARNet) for the RUL prediction of rolling bearings. Firstly, a cross-channel maximum pooling layer was designed to automatically select the more important information. Secondly, a lightweight feature reuse multi-scale attention unit was developed to extract the multi-scale degradation information in the vibration signals and recalibrate the multi-scale information. Then, end-to-end mapping between the vibration signal and the RUL was established. Finally, extensive experiments were used to demonstrate that the proposed FRMARNet model can improve prediction accuracy while reducing the number of model parameters, and it outperformed other state-of-the-art methods.

## 1. Introduction

New industrial paradigms, including network collaborative manufacturing, industrial big data, and cyber-physical systems, have promoted the transformation of traditional manufacturing to intelligent manufacturing [1]. High accuracy, high reliability, and high efficiency are the main development directions of rotary machinery health monitoring against the background of intelligent manufacturing [2]. Various sensors installed on rotating machinery equipment can collect massive data, which contains a large amount of information related to equipment degradation. Therefore, big data provides new opportunities and challenges for rotating machinery health monitoring. Rotating machinery structures are becoming more complex and operate in harsh environments for long periods of time [3,4]. Rolling bearing is one of the most important components in rotating machinery whose abnormal state and fault may lead to equipment shutdown, resulting in huge economic losses and even safety accidents [5]. Therefore, to achieve predictive maintenance and improve the reliability of rotating machinery, it is necessary to carry out remaining useful life (RUL) prediction for the intelligent health monitoring of rolling bearings. RUL prediction is defined as an estimate of time to failure according to the International Organization for Standardization [6].

The RUL prediction methods of rolling bearings are mainly divided into model-based methods, data-driven methods, and hybrid methods [7]. The model-based method is based on the degradation mechanism of rolling bearings and relies on expert experience and prior knowledge to establish a physical model that can accurately reflect the changing trend of RUL. However, modern mechanical equipment is becoming more and more complex, and it is difficult or even impossible to establish such a physical model [8], the model-based approach is difficult to popularize in industrial applications. With the development of sensor technology and data transmission technology in recent years, data-based methods have developed rapidly [9]. The data-based method collects the monitoring data of rolling bearings, and then the machine learning (ML) model is adopted to establish a nonlinear mapping between easily obtained monitoring signals and difficult-to-obtain RUL values. This approach is mainly based on a large amount of data acquired by sensors and requires less inherent degradation mechanism and prior knowledge of the system. Hybrid methods are still based on rolling bearing degradation mechanisms and have the same disadvantages as physics-based methods. It is still challenging to establish effective hybrid models.

Among the above three approaches, data-driven methods have the advantages of simple implementation, fast response, high prediction accuracy, less prior knowledge, and expert experience, and they are conducive to deployment in real industrial applications. Therefore, data-driven methods have attracted the interest of many researchers in academia and industry [7]. Data-based methods can be further divided into traditional ML-based methods and deep learning-based methods. The traditional ML-based method is mainly divided into four steps: data acquisition, manual feature extraction and selection, model training, and RUL prediction [8,10]. However, there are two main problems with traditional ML methods. Firstly, with traditional ML methods, it is difficult to extract deep features from massive data and they cannot make full use of the advantages of big data [11]. Secondly, manual feature extraction and selection is time-consuming and challenging. The vibration signal of rolling bearings exhibits non-stationary and complex characteristics [12]. The degradation trend of mechanical equipment performance cannot be accurately obtained by artificial feature information extraction methods, such as information entropy [13]. It is not satisfactory to adopt information theory (such as information entropy) to realize RUL prediction for complex vibration signals. Deep learning (DL) can automatically and adaptively extract key feature information from vibration signals, which can well replace artificial feature information extraction based on methods such as information entropy or signal processing. Therefore, the lack of large-scale data processing and automatic feature information extraction capabilities limits the application of traditional ML methods in modern industry.

To deal with the above shortcomings, DL was proposed by Hinton in 2006 [14]. DL can automatically extract discriminative deep features from raw data by superimposing deep hidden layers and nonlinear transformation [15]. Therefore, in terms of processing flow, the most significant difference between DL-based methods and traditional ML-based methods is that automatic feature extraction replaces manual feature extraction. The DL model can automatically learn a set of optimal parameters for feature extraction without manual feature selection. The powerful feature extraction ability promotes DL to achieve promising performance in rotating machinery condition monitoring [16], fault diagnosis [17,18], and RUL prediction [5,19]. Many scholars have adopted DL technologies, such as auto-encoder [20], vanilla convolutional neural networks (CNNs) [21], residual networks (ResNets) [22], recurrent neural networks (RNNs) [23], and transformers [24] for RUL prediction. The methods based on DL have been widely used in the prediction of rolling bearing RUL. Specifically, CNN takes convolution operation as the core, the characteristics of sparse connection, and weight sharing to reduce the parameter number to be trained and the computational burden. The identity mapping of ResNet is beneficial for stacking deeper models for extracting deep features. The powerful feature extraction ability and efficient network training process contribute a CNN-based method to the most widely used model for RUL prediction of rolling bearings. In general, the convolution operation adopts a fixed convolution kernel for feature extraction. For convolution operation, a large convolution kernel is preferred to extract a large range of global information, while a small convolution kernel is preferred to extract a small range of local information. Therefore, the use of a fixed convolution kernel will inevitably lose feature information and lead to a decline in prediction accuracy. Researchers have developed many multi-scale CNN models for RUL prediction. Li et al. [9] adopted three parallel branches with different convolution kernels to extract multi-scale features in the raw data and then aggregated the features with a sum for RUL prediction. In the studies [2,25], multiple convolution kernels of different sizes have extracted features in parallel, and the sum of feature maps has been obtained after weighted processing for fault diagnosis. In addition, some other researchers have aggregated multi-branch feature information by concatenation. In the studies [26,27,28,29,30,31,32], convolution kernels of different sizes have been adopted to extract features in parallel and then concatenated together. However, the above multi-scale CNN methods for the RUL prediction of rolling bearings have two obvious disadvantages. 

(1) Existing multi-scale CNN methods adopt different convolutional kernel branches to extract multi-scale features in parallel and then simply aggregate the feature maps by addition or concatenation. This method will significantly increase the parameter number of the model, which results in serious computational burden and parameter optimization difficulties. (2) Since the degradation features of rolling bearings may be distributed at different ranges at different stages of their life cycle, the features extracted by multibranch parallelization are redundant. Different convolutional channels may contain feature information of different scales. The existing methods lack cross-channel feature interaction and an explicit learning mechanism to efficiently distinguish the importance of feature information contained in different channels. 

To tackle the aforementioned shortcomings, we propose an extremely lightweight DL model for the RUL prediction of rolling bearings, the so-called feature reuse multi-scale attention residual network (FRMARNet). In the proposed FRMARNet, a novel cross-channel feature interaction layer is first constructed to extract the more important feature information. Then, a feature reuse multi-scale attention (FRMA) learning strategy is developed by partitioning channels into groups, and the current group reuses the features of the previous group, which efficiently captures the features of various scales without increasing the model parameters. In the FRMA unit, the attention module is constructed to emphasize more important scale information. Finally, the learned high-level degradation features are fed into the regression layer to predict the RUL. A bearing RUL prediction experiment was conducted to evaluate the effectiveness and superiority of the proposed method. The experimental results indicate that the developed framework can obtain better prediction performance, and the accuracy was superior to other DL models. The main contributions of this paper can be summarized as follows. 

(1) A cross-channel feature interaction mechanism is proposed to automatically select important feature information without increasing the model parameters and reducing the impact of low-value redundant information on subsequent network feature extraction. 

(2) A new extremely lightweight FRMA unit is proposed for the RUL prediction of rolling bearings, which consists of a feature reuse multi-scale module and a multi-scale feature adaptive calibration module.

(3) FRMARNet was constructed using a feature interaction layer and by stacking multiple FRMA units. Extensive experiments were conducted to evaluate the developed method. 

The rest parts of this paper are organized as follows. In the upcoming section, the theoretical background of ResNet and multi-scale CNN is presented. Section 3 details the proposed FRMARNet. In Section 4, bearing run-to-failure datasets are used to evaluate the effectiveness of the proposed method in improving the accuracy of RUL prediction. Finally, the conclusions are given in Section 5.

## 2. Theoretical Background

In this section, the basic structure of ResNet is presented, and then the basic principle of multi-scale CNN is introduced.

### 2.1. Basic Structure of ResNet

**Figure 1:**
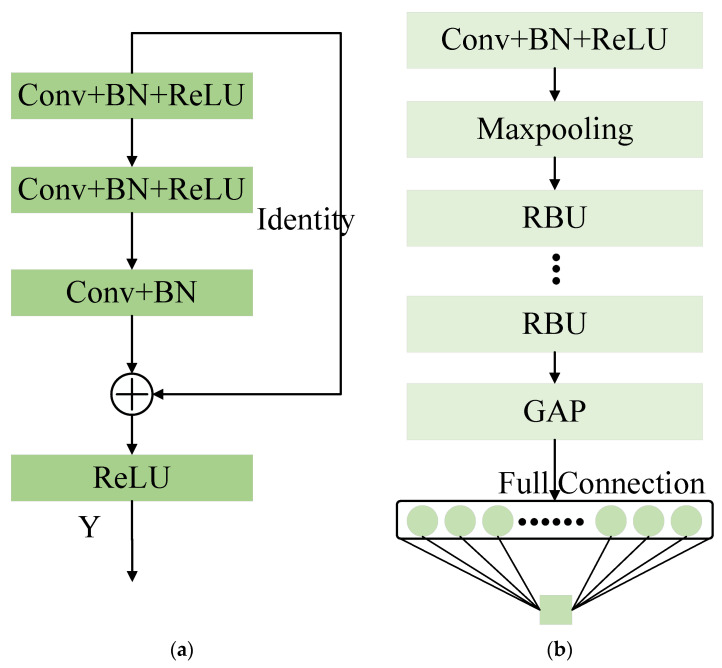
A brief architecture of RBU and ResNet: (**a**) RBU; (**b**) ResNet.

In recent years, ResNet has become the most popular DL method for the RUL prediction of rolling bearings. The major superiority of ResNet is that it can still optimize parameters well when the model is deeper in layers [33]. Specifically, as described in reference [33], the training error of a 56-layer convolution is higher than that of 20 layers on the CIFAR-10 dataset. With the network depth increasing, the accuracy becomes saturated and then degrades rapidly. The model adds more layers to a suitable depth, which leads to higher training errors. ResNet adds identity mapping between networks to optimize model training. The entire network can be optimized end-to-end by stochastic gradient descent (SGD) with error backpropagation. The typical structure of a residual building unit (RBU) and ResNet is shown in Figure 1. The RBU is the core component of ResNet, which is composed of multiple convolution (Conv) layers, batch normalization (BN) [34] layers, and rectified linear unit (ReLU) activation function layers. ResNet is constructed by stacking Conv + BN + ReLU, a maxpooling layer, multiple RBUs, a global average pooling (GAP) layer, and a fully connected layer.

The convolution layer extracts the feature matrix by the convolution operation. The convolution operation is performed by sliding a convolution kernel of a certain size over the data matrix and multiplying the values at the corresponding positions to sum the results to form a new feature matrix. Compared to the traditional full-connection neural network, sparse connection and weight sharing contribute a CNN that is relatively easy to train. The convolution operation can be expressed as [35]:

(1) $$y_{j}^{c}=ReLU(BN(b_{j}^{c}+\sum_{m=1}^{M}w_{m,j}^{c}⊗x_{m}^{c-1}))$$

where $y_{j}^{c}$ is the jth channel output feature map of the cth convolutional layer, $b_{j}^{c}$ is the bias of the jth feature map, $w_{m,j}^{c}$ denotes the weight matrix of the convolution kernel; m denotes the number of input channels; ⊗ denotes the convolution operation; and $x_{m}^{c-1}$ is the mth channel input feature map of the cth convolutional layer.

BN is an adaptive normalization method used to reduce the internal covariance shift problems and accelerate deep network training, making the feature distribution more stable across batches. BN can be formulated as follows [34]:

(2) $$\mu_{B}=\frac{1}{m}\sum_{i=1}^{m}x_{i}$$

(3) $$\sigma_{B}^{2}=\frac{1}{m}\sum_{i=1}^{m}{\left(x_{i}-\mu_{B}\right)}^{2}$$

(4) x^i=xi−μBσB2+ϵ

(5) yi=γx^i+β

where m denotes the number of samples contained in each batch $;x_{i}$ is the data of the ith sample in a batch; $\mu_{B}$ and $\sigma_{B}$ denote the mean and variance of a batch, respectively; ϵ denotes a constant very close to zero; $y_{i}$ is the output after passing through the BN layer; γ and β are two trainable parameters by gradient descent to optimize the adaptive normalization method.

Nonlinear transformation is implemented in ResNet using the ReLU activation function. The derivative of the ReLU activation function can only be 1 or 0, which helps to alleviate the problem of gradient disappearance and gradient explosion. The output of ReLU is max(x,0). 

An RBU consists of three convolutional layers, three ReLU activation function layers, three BN layers alternately combined, and an identity shortcut, as shown in Figure 1a. The output of RBU can be expressed as [33]:

(6) $$Y=ReLU(F\left(x\right)+x)$$

where x denotes the input of the RBU; $F\left(x\right)$ is the output of the third convolutional layer in the RBU. The identity shortcut improves the efficiency of the parameter optimization, allowing ResNet to outperform the vanilla CNN.

GAP is used before the fully connected layer to significantly reduce the feature dimension, thus reducing the number of parameters in the fully connected layer, which is beneficial for improving training efficiency and preventing overfitting. Given the input $x\in R^{I\times J\times C}$, I, J and C represent the row, column, and channel spatial dimension of the input matrix x. The output $y_{c}$ of the cth channel after the GAP operation can be expressed as:

(7) $$y_{c}=\frac{1}{I\times J}\sum_{\begin{array}{c} 1\leq i\leq I\\ 1\leq j\leq J \end{array}}x(i,j,c)$$

### 2.2. Multi-Scale CNN

**Figure 2:**
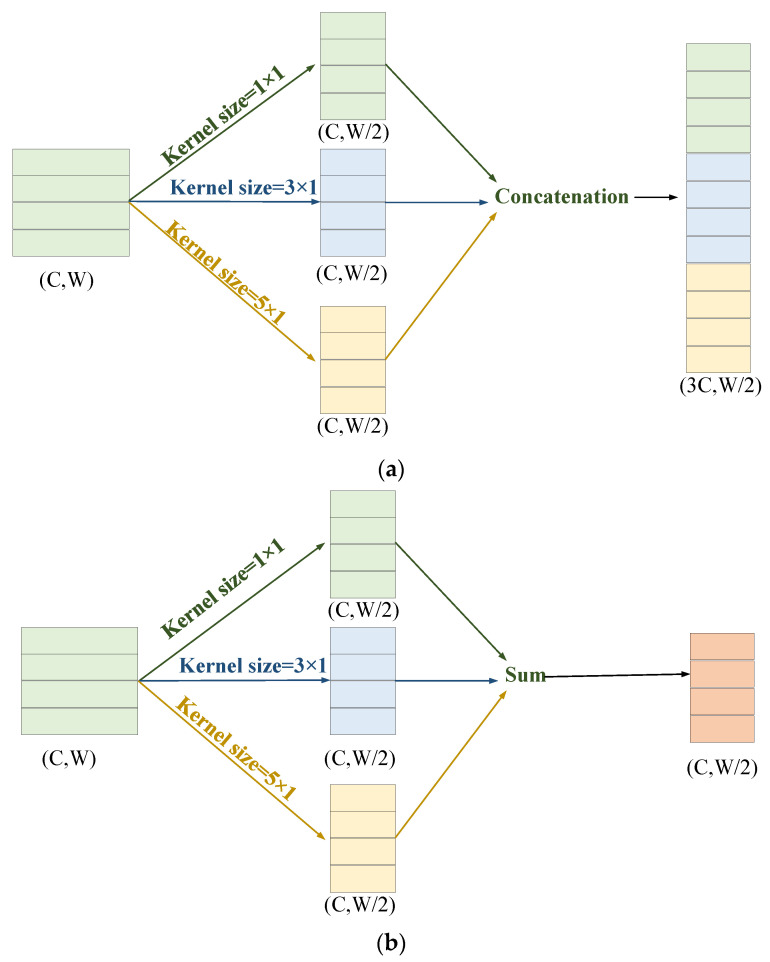
Diagram of the multi-scale convolutional aggregation features: (**a**) soncatenation; (**b**) sum.

The size of the convolution kernel is an important parameter of CNNs, and different sizes of the convolution kernel can extract features from different time scales. Specifically, the monitoring signals of rotating machinery may contain less degradation feature information at the early stage of equipment degradation, so these degradation features are distributed over a larger time range. In the late stage of equipment degradation, equipment wear becomes more serious, and the probability of failure greatly increases. The monitoring signal may contain more degradation feature information, so this feature information may be distributed in a small time range [31]. According to the way in which different convolution kernel branch operation results are aggregated, they can be divided into concatenation and sum modes. The concatenation mode aggregates the feature maps of different convolution kernel branches through channel concatenation, as shown in Figure 2a. The sum mode aggregates the feature maps of different convolution kernel branches through element-wise summation, as shown in Figure 2b. Note that the stride of the convolution operation is set to as 2.

Existing multi-scale methods have significantly increased the parameter numbers in the model, thus imposing a heavy computational and parameter optimization burden. Taking one-dimensional RBU as an example to compare the number of parameters of the vanilla CNN (VCNN), the sum multi-scale CNN (SMCNN), and the concatenation multi-scale CNN (CMCNN), the parameter quantities for each model are calculated as:

(8) $$P_{VCNN}=CC_{1}K_{1}+C_{1}C_{2}K_{2\_2}+C_{2}C_{3}K_{3}$$

(9) $$P_{SMCNN}={CC}_{1}K_{1}+(C_{1}C_{2}K_{2\_1}+C_{1}C_{2}K_{2\_2}+C_{1}C_{2}K_{2\_3})+C_{2}C_{3}K_{3}$$

(10) $${P_{CMCNN}=CC}_{1}K_{1}+(C_{1}C_{2}K_{2\_1}+C_{1}C_{2}K_{2\_2}+C_{1}C_{2}K_{2\_3})+{3C}_{2}C_{3}K_{3}$$

where $P_{VCNN}$, $P_{SMCNN}$, and $P_{CMCNN}$ represent the number of parameters for the three different models, respectively. C represents the number of input channels. $C_{i}$ and $K_{i}$ represent the number of convolution channels and kernel size in the ith layer, respectively. $K_{2\_j}$ represents the jth kernel size in the multi-scale convolution. It is obvious that $P_{CMCNN}>P_{SMCNN}>P_{VCNN}$, and as the number of convolution layers increases, $P_{CMCNN}$ and $P_{SMCNN}$ will be significantly larger than $P_{VCNN}$. Therefore, although multi-scale CNNs can improve model prediction accuracy to a certain extent in theory, they also bring many parameters, which leads to increased difficulty in parameter optimization and increased risk of overfitting.

## 3. The Proposed RUL Prediction Method

This section introduces in detail the proposed FRMARNet model. The proposed FRMARNet utilizes raw monitoring data as the input. Firstly, a cross-channel maximum pooling (CMP) is proposed to automatically select important feature information. Secondly, to extract more important multi-scale features, the FRMA unit is developed to capture multi-scale information in the data and recalibrate the importance of the feature information. Note that the proposed FRMARNet model is a lightweight approach; the CMP layer and FRMA unit hardly increase the number of parameters.

### 3.1. Feature Reuse Multi-Scale Attention Residual Network

(1) Cross-channel maximum pooling

**Figure 3:**
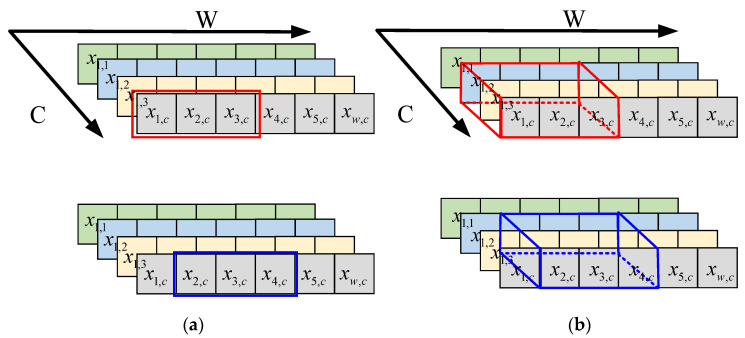
Diagram of the two pooling methods: (**a**) one-dimensional maximum pooling; (**b**) CMP.

In the high-dimensional features extracted by deep CNN models, larger values contain more important feature information, and smaller values contain more redundant or even noisy information. The maximum pooling operation is based on this idea used in CNN for feature dimensionality reduction and the removal of redundant information. The maximum pooling is on each channel, and the maximum value in the pooling region is selected as the output, as shown in Figure 3a, where the red area represents the first pooling block, the blue area represents the second pooling block with a sampling interval of 1, C represents the number of channels, and W represents the data width. The one-dimensional maximum pooling can be expressed as:

(11) $$p_{w,c}=max\{x_{\left(w-1\right)\times s+m,c}\}$$

where $p_{w,c}$ is the value of the pooling layer output at coordinates (w,c), s represents the sampling interval of pooling; c represents the cth channel, and w represents the wth data point. $x_{\left(w-1\right)\times s+m,c}$ is the value of the input feature map at coordinates ((w−1)×s+m,c), where m∈[1,k], k represents the size of the pooling region.

For the original one-dimensional signal, the high-dimensional features obtained by a convolution operation are fed into the one-dimensional pooling operation to highlight the important feature information. However, this approach does not consider the effect of features between different channels. Cross-channel interactions are often mapped as new combinations of features that consider the interaction of features between different channels. Cross-channel feature interactions have been used in attention mechanisms [36,37] and have significantly improved the ability of DL models to extract important feature information. Inspired by the idea of cross-channel interaction, without increasing the number of model parameters, this paper proposes a CMP operation based on one-dimensional maximum pooling, as shown in Figure 3b. CMP can be expressed as:

(12) $$p_{w,c}=max\{x_{\left(w-1\right)\times s+m,\left(c-1\right)\times s+n}\}$$

where m,n∈[1,k]. Assuming the size of the pooling area is 3 and the number of cross-channels is 3, CMP selects the maximum of nine positions as the output. Compared to the original maximum pooling operation, CMP not only considers the feature information of adjacent positions of the same channel but also considers the feature information of adjacent channels, which is helpful for the network to extract more important feature information.

(2) Feature reuse multi-scale attention unit

**Figure 4:**
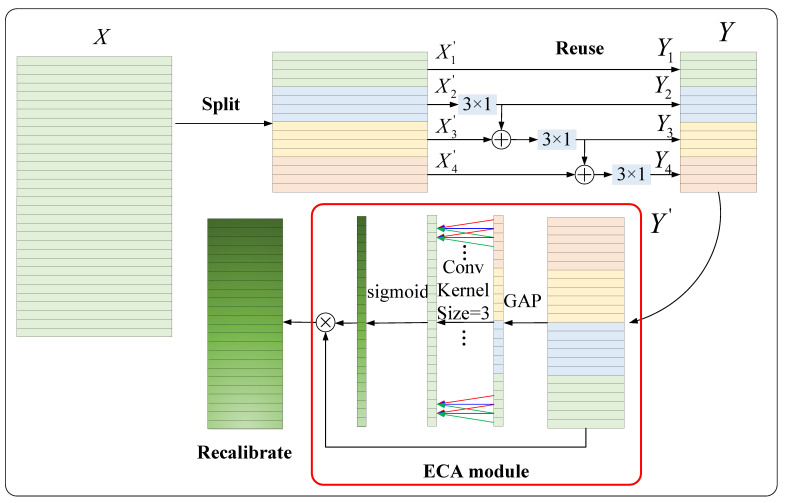
Diagram of the proposed FRMA unit.

To capture the multi-scale degradation information in the signal without increasing the parameters and to recalibrate the importance of multi-scale information, an improved Res2Net [38] block, the so-called FRMA unit, is proposed in this paper. Unlike the original Res2Net, an attention mechanism is added to the FRMA unit to recalibrate the importance of the feature information at different scales, thus highlighting important scale information and suppressing redundant scale information. Specifically, we implement the FRMA unit via three steps, as illustrated in Figure 4. In Y, different colors represent different groups. The red box shows the attention mechanism. The detailed implementation of FRMA is described as follows.

Split: First, to reduce the number of parameters in multi-scale feature extraction, the convolution operation with a kernel size of 1×1 is adopted for channel dimensionality reduction. An excessive channel dimensionality reduction ratio will result in the loss of important feature information, while a small channel size reduction rate is insufficient to compress feature information and is not conducive to parameter reduction. The channel dimension reduction ratio is an empirical hyperparameter and set as 2, which follows the settings in references [33,38]. The given feature map $X\in R^{C\times W}$ is transformed to $X^{\prime }\in R^{(c/2)\times W}$. Then, the input channels are split into groups, $X^{\prime }=\left[X_{1}^{\prime },X_{2}^{\prime },X_{3}^{\prime },\ldots \ldots {,X}_{s}^{\prime }\right]$, where s represents the number of groups, and $X_{i}^{\prime }$ represents the feature map subsets, i∈[1,2,3,……,s]. In Figure 4, a four-group case is shown.

Reuse: As mentioned in the previous section, our goal is to achieve multi-scale feature extraction without increasing the number of parameters. In the well-known InceptionV3 network [39], a serial structure with two convolutional kernels of 3×3 can equivalently obtain scale information of 5×5. To achieve this goal, the authors of this paper adopted a hierarchical structure of feature information reuse. The basic idea is to reuse feature map subsets from different groups. Specifically, the former group of features that have been convolved and the next group of features that have not been convolved are fused and then sent into the convolution operation. After multiple processing steps, the model can obtain information at different scales. The output of the feature map subsets $X_{i}^{\prime }$ can be expressed as:

(13) $$Y_{i}=\left\{\begin{array}{cc} X_{1}^{\prime }, & i=1\\ RBC\left(X_{2}^{\prime }\right), & i=2\\ RBC\left(X_{i}^{\prime }+Y_{i-1}\right), & 3\leq i\leq s \end{array}\right.$$

where $Y_{i}$ denotes the output of the feature map subsets $X_{i}^{\prime }$; RBC(·) represents the tandem connection of the one-dimensional convolutional layer with a kernel size of 3×1, a BN layer, and a ReLU activation function layer. It can be seen in Equation (13) that when 3≤i≤s, the input to $Y_{i}$ is the sum of the sub-feature map $X_{i}^{\prime }$ and $Y_{i-1}$. Taking $Y_{3}$ as an example, $Y_{2}$ obtains the receptive field of 3×1, so ${(Y}_{2}$+$X_{2}^{\prime })$ also contains the receptive field of 3×1, and the next convolution operation with a kernel size of 3×1 can obtain the receptive field of 5×1. Similarly, $Y_{4}$ can obtain a receptive field of 7×1. Finally, $Y_{i}$ is concatenated together to form a new feature map Y. This grouped hierarchical structure of feature information reuse not only extracts multi-scale information and enhances the feature interaction between channels but also further reduces the number of model parameters. The parameter quantities of the FRMA unit are calculated as:

(14) $$P_{FRMA}=CC_{1}K_{1}+{((C}_{1}/s)LK_{2\_2})s+LsC_{3}K_{3}=CC_{1}K_{1}+C_{1}LK_{2\_2}+LsC_{3}K_{3}$$

where L represents the number of channels in the feature map $Y_{i}$, $Ls=C_{2}$. It can be seen in Equation (14) that FRMA significantly reduced the parameters compared to the vanilla CNN and the multi-scale CNN in Section 2.2.

Recalibrate: First, to better fuse information at different scales and enrich the feature information, the convolutional operation with a kernel size of 1×1 is adopted to achieve channel dimensionality increase. An excessive channel dimension increase ratio will bring too many parameters to subsequent convolution operations. The channel dimension increase ratio is an empirical hyperparameter and set as 4, which follows the settings in references [33,38]. The feature map $Y\in R^{\left(Ls\right)\times \left(W/2\right)}$ is transformed to $Y^{\prime }\in R^{\left(4Ls)\times (W/2\right)}$. The feature information Y extracted by the feature reuse multi-scale module contains multi-scale information and a large amount of redundant information. The authors of this paper adopted the efficient channel attention (ECA) mechanism [37] for adaptive prompting of the modules to focus on the scale information related to device degradation and to weaken redundant information. As shown in Figure 4, the components in the red box form the ECA mechanism module. The input $Y^{\prime }$ passes through the GAP layer, 1D convolution layer, and sigmoid activation function layer in turn to obtain the weights of different channels. Specifically, the global information of each layer is first obtained through GAP. Then, the channel-wise dependencies are captured by 1D convolutional operations with a fixed kernel size of 3 × 1. The ECA module captures local cross-channel interaction by considering every channel and its 3 neighbors. Note that the 1D convolution with a kernel size of 3 × 1 and channel 1 brings a negligible amount of 3 parameters. Therefore, unlike the squeeze-and-excitation (SE) attention module [36], the ECA module is a lightweight plug-and-play unit. Thirdly, the simple gating mechanism with a sigmoid activation is employed to generate the corresponding channel weights ω. Finally, the output $\hat{Y}$ refers to channel-wise multiplication between the input $Y^{\prime }$ and the channel weights ω. The cth channel of $\hat{Y}$ is calculated by:

(15) $$\hat{Y}\left(c\right)=Y^{\prime }\sigma (Conv(g(Y^{\prime })))$$

where g represents GAP, Conv represents a convolutional operation with a fixed kernel size of 3×1, and σ represents the sigmoid activation function.

### 3.2. RUL Prediction Based on FRMARNet

**Figure 5:**
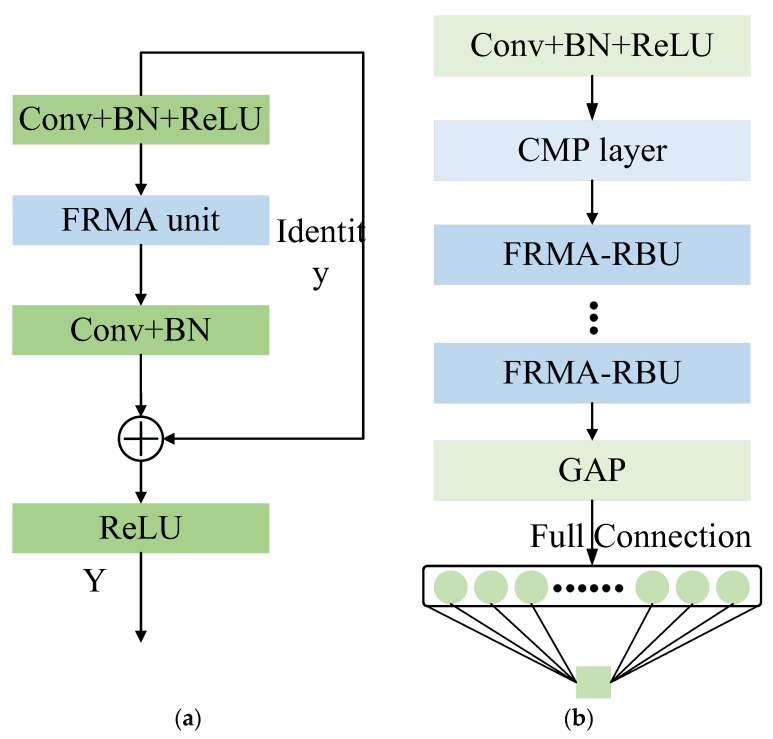
Architecture of the developed FRMARNet: (**a**) FRMA-RBU module; (**b**) FRMARNet.

The core building block of FRMARNet can be established using the FRMA unit and an RBU. As shown in Figure 5a, the new multi-scale attention residual module FRMA-RBU can be constructed by replacing the second Conv+BN+ReLU layer in the original RBU with an FRMA unit. As shown in Figure 5b, the FRMARNet model is constructed by stacking the Conv+BN+ReLU layer, CMP layer, and multiple FRMA-RBU modules in sequence, which is beneficial to learn valuable high-level multi-scale features from the raw vibration data. RUL prediction can be achieved by feeding the learned features into the GAP and FC layers. In terms of implementation details, compared to Figure 1, the CMP layer replaces the maximum pooling layer, and the FRMA-RBU module replaces the RBU module in the original ResNet model.

The regression layer consisting of a full connection is used to predict the RUL. Gradient descent and error backpropagation are employed to train the FRMARNet model. Given a training dataset $D={\left\{x_{i},y_{i}\right\}}_{i=1}^{n}$ with n samples, the developed FRMARNet utilizes the root mean square error (RMSE) loss function to optimize the parameters, which is suitable for the regression problem. RMSE can be expressed as:

(16) Ly^,y;θ=1n∑i=1n(yi−y~i)2

where $y_{i}$ and ${\tilde{y}}_{i}$ denote the true and predicted values, respectively. Ly^,y;θ represents loss function. $\theta =\left\{W,b\right\}$ represents the trainable parameters in the FRMARNet, which can be updated and optimized by Adam with error backpropagation:

(17) $$W^{*}\leftarrow W-\eta (\frac{\partial L}{\partial W})$$

(18) $$b^{*}\leftarrow b-\eta (\frac{\partial L}{\partial b})$$

where η is the learning rate. On the basis of SGD, many parameter optimizations have been developed. RMSprop can be considered an enhancement of SGD. Combining momentum and RMSprop, Adam was identified as a desirable method for dynamically adjusting the learning rate [40]. In order to obtain the optimized model, Adam was selected as the optimizer in this paper. The momentum was set as 0.9. The training steps and parameter optimization of FRMARNet are presented in Algorithm 1.
**Algorithm 1:** training of FRMARNet**Input:** Training dataset: $D={\left\{x_{i},y_{i}\right\}}_{i=1}^{n}$; Learning rate: η; Batch size: $n_{b}$; Max-epoch:E; Momentum = 0.9. 1: Initialize trainable parameters $\theta =\left\{W,b\right\}$ of FRMARNet 2: **for** epoch = 1, 2, 3,……, Max-epoch **do** 3:    **for** step = 1, 2, 3,……, Max-step **do** 4:         //Feature extract 5:         Conv+BN+ReLU module; 6:         CMP module; 7:         FRMA-RBU module; 8:         GAP module; 9:        //regressor 10:        Feed into full connection and obtain $\tilde{y}$; 11:       //Calculate loss and gradient descent 12:        Calculate RMSE loss Ly^,y;θ; 13:        Calculate gradient and update parameters, 14:        $W^{*}\leftarrow W-\eta (\frac{\partial L}{\partial W})$, $b^{*}\leftarrow b-\eta (\frac{\partial L}{\partial b})$. 15:    **end for** 16: **end for** **Output:** Optimized weights and biases $\theta^{*}=\{W^{*},b^{*}\}$


**Figure 6:**
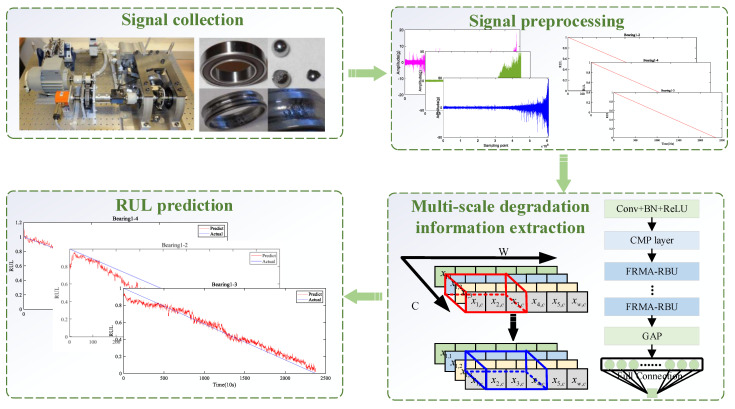
The RUL prediction procedure of the proposed FRMARNet method.

Based on the proposed FRMARNet, we present a new RUL estimation framework as follows. The framework includes four steps: signal collection, signal preprocessing, multi-scale degradation information extraction, and RUL prediction, as shown in Figure 6. The signal collection part comes from the PRONOSTIA platform [41]. 

Step 1: Collecting rolling bearing run-to-failure vibration dataset by conducting accelerated degradation experiments.

Step 2: Multiple rolling bearings are divided into training and testing sets. Some of the bearings are used for training, while the others are used for testing. The vibration signal of each rolling bearing is divided into multiple samples, tagging samples with RUL values to construct run-to-failure datasets. The principle of tagging samples with RUL values is explained in Section 4.2.

Step 3: After determining the relevant hyperparameters and initializing the network weights, the training data are fed into the constructed FRMARNet for multi-scale degradation information. This step enables offline training of the DL model.

Step 4: The trained model is deployed online and used on test data for RUL prediction. The prediction results can be used for the predictive maintenance of rotating machinery.

## 4. Experimental Verifications

To validate the proposed method in dealing with the RUL prediction of rolling bearings, a case study was conducted to evaluate the prediction performance of FRMARNet in this section.

### 4.1. Experimental Platform

**Figure 7:**
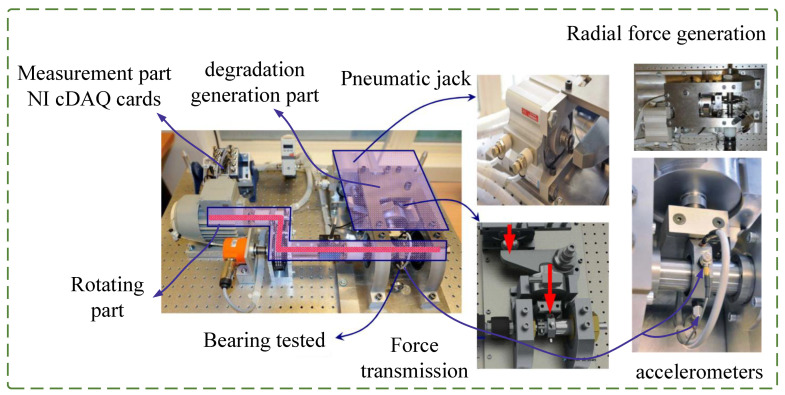
Experimental platform for collecting bearing degradation data.

Bearing real run-to-failure degradation data from the PRONOSTIA platform [41] were employed for experimental verification. The main objective of the experimental platform is to provide realistic experimental data to characterize the degradation of ball bearings throughout their whole operational life. As shown in Figure 7, the experimental platform consisted of three parts: the rotating part, the degradation generation part, and the measurement part. The rotating part mainly included an asynchronous motor and gearboxes. The motor generated 250 W of power and transmit the rotating motion to drive the bearing through the gearboxes. The degradation generation part was the core of the whole experimental platform. The radial force generated by the jack acted on the tested bearing. Radial forces reduced the bearing life by setting the value to the maximum dynamic load of the bearing (4000 N). The measurement part mainly included an NI data acquisition device and accelerometers. The accelerometers were placed on the outer ring of the bearing to measure degradation data in both the horizontal and vertical directions. The degradation data sampling frequency was 25.6 kHz, the sampling time was 0.1 s, and the sampling interval was 10 s. In other words, 2560 numbers in 0.1 s were collected every 10 s. Therefore, each sample contained 2560 numbers and an RUL label.

### 4.2. Dataset Description

Under the working conditions of a motor speed of 1800 r/min and a load of 4000 N, the real run-to-failure degradation data of seven bearings were collected. These bearings are of the same type and dimensions. The characteristics of the tested bearings are shown in Table 1. Horizontal vibration signals from the accelerometer were used in the RUL prediction studies. The details of the used dataset are shown in Table 2; different bearings had different accelerated degradation lifetimes and sample lengths. The longest experiment duration was bearing1_1, with 28,030 s, and the shortest experiment duration was bearing1_2, with 8710s. There were 14,647 sample instances in total, and each sample instance contained 2560 data points. To comprehensively evaluate the performance of the model, the leave-one-out cross-validation strategy [24,42] was utilized for each test. That is, during the cross-validation process, while one bearing was used for testing, the other six bearings were used for training. For example, the dataset of bearing1_2–bearing1_7 was used as the training data and that of bearing1_1 as the testing data in the first cross-validation. 

The health indicator is a custom indicator that helps researchers to evaluate the health status of machinery in real time [10]. The health indicator is also the label corresponding to each sample in RUL prediction. In physical model-based RUL prediction, it is critical to construct an appropriate health indicator through expert experience and prior knowledge. In the data-based approach, to reduce the manual and empirical process, a linear health indicator representation was utilized [24,42]. Specifically, the RUL percentages were calculated as the health indicator to evaluate the health status of the bearings. The RUL label corresponding to the sample was converted into the RUL percentage for training. Assuming that the sample serial numbers collected during the whole life of the bearing are 1,2,3,⋯,i in sequence, the health indicator of the ith sample in any stage can be calculated by:

(19) $${RUL}_{i}=\frac{n-1-i}{n-1}$$

where ${RUL}_{i}$ represents the health indicator of the ith sample, and n represents the total number of samples. Note that the health indicator was confined in the range of [0, 1], where the larger the indicator value, the healthier the bearing state. The smaller the indicator value, the closer the bearing is to failure. Specifically, taking bearing1_1 as an example, a health indicator equal to 1 means that the bearing is in a brand-new state, and the real RUL is 28,030 s. A health indicator equal to 0.5 means that the real RUL is 14,015 s, and a health indicator equal to 0 means the bearing has failed, and the real RUL is 0.

### 4.3. Evaluation Metrics

To quantitatively evaluate the predictive performance of the proposed FRMARNet model, the RMSE and mean absolute error (MAE) were utilized as the evaluation metrics, which can be calculated by:

(20) $$RMSE=\sqrt{\frac{1}{n}\sum_{i=1}^{n}{\left(y_{i}-{\tilde{y}}_{i}\right)}^{2}}$$

(21) $$MAE=\frac{1}{n}\sum_{i=1}^{n}\left|y_{i}-{\tilde{y}}_{i}\right|$$

where $y_{i}$ and ${\tilde{y}}_{i}$ denote the true and predicted health indicators, respectively, and n represents the total number of samples. Smaller RMSE and MAE values represent better prediction performance. To further reduce the deviation between the predicted and real values, and to make the prediction results better reflect the equipment degradation trend, a weighted average method was employed to smooth the prediction result, as described in the literature [42].

### 4.4. Model Structure and Hyperparameter Configurations

In this section, the FRMARNet is established for RUL prediction, and we will determine the proposed DL model structure and hyperparameters. The architecture of the developed model is listed in Table 3, where C(16×64×2) represents that the convolutional kernel size is 64×1 and stride is 2 with 16 output channels. P(3×2) represents that the size of the CMP layer pooling region is 3, cross-channel number is 3, and the sampling interval is 2. For FR(64−2L×2×s−128), 64 represents the first convolutional kernel size is 1×1, stride is 1, and output channel is 64 in the FRMA-RBU module. 2L×2×s represents the number of convolutional channels in the feature map $Y_{2}$ is 2L, stride is 2, and groups is s in the FRMA-RBU module. The number 128 represents the third convolutional kernel size is 1×1, stride is 1, and output channel is 128 in the FRMA-RBU module. The values of L and s will be determined by experimental studies in Section 4.5. G(40×1) represents the GAP pooling region is 40×1. FC(1) denotes that the final output is 1, which is a fixed value in a regression problem. The input feature size (1252×16) represents the input data width, which is 1252, and the input data channel, which is 16 of the layer. Output feature size (626×8) means the output data width is 626 and the output data channel is 8 of the layer.

In addition, the other hyperparameters in DL that are relevant for model training are obtained empirically. The batch size was set to 256, indicating that 256 samples were randomly selected for model training in each iteration. The learning rate was set to 0.00003 for bearing1_1 and bearing1_3–bearing1_7 and 0.000005 for bearing1_2. The Adam optimization algorithm was selected as the optimizer of the FRMARNet model. Finally, the experiment was iterated for a total of 120 epochs.

### 4.5. RUL Estimation

(1) RUL prediction results

The RUL prediction experiment was carried out in Windows, i7 CPU, and RTX 2060 SUPER GPU. The program was developed in a Python environment based on the PyTorch framework. Each experiment was repeated five times to reduce the influence of the randomization of the initial parameters. The average values of the evaluation metrics were used as the final evaluation. Bearings1_1–bearing1_7 were all used for testing. The number of channels L and the number of groups s are two important hyperparameters of the feature reuse multi-scale module. When L is fixed, a larger s contains richer scale information, but it may bring too much redundant information. A smaller s contains less scale information, the degradation information in the data may not be completely extracted. When s is fixed, the larger L is, the larger the number of parameters will be, which may reduce the precision. This may be due to overfitting caused by many parameters. An L value that is too little will not extract enough representative features. 

**Figure 8:**
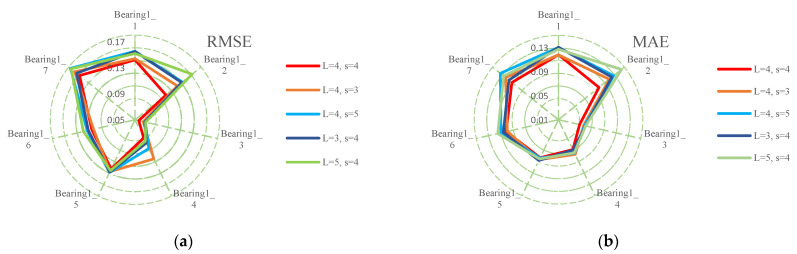
The accuracy comparison of different hyperparameters: (**a**) RMSE; (**b**) MAE.

The authors of this paper compared five different combinations, and the experimental results are shown in Table 4 and Table 5 and Figure 8. When fixing L, the lowest RMSE and MAE were achieved when s=4 in all bearings except bearing1_6. The lowest RMSE and MAE were achieved when s=3 in bearing1_6. When fixing s, the lowest RMSE and MAE were achieved when L=4 in all bearings. This phenomenon indicates that s and L values that are too large bring too much redundant information and parametric quantities, which is not conducive to extracting important feature information and parameter optimization. s and L values that are too small do not fully extract important feature information. The experimental results show that L=4 and s=4 are the optimal hyperparameter combination.

**Figure 9:**
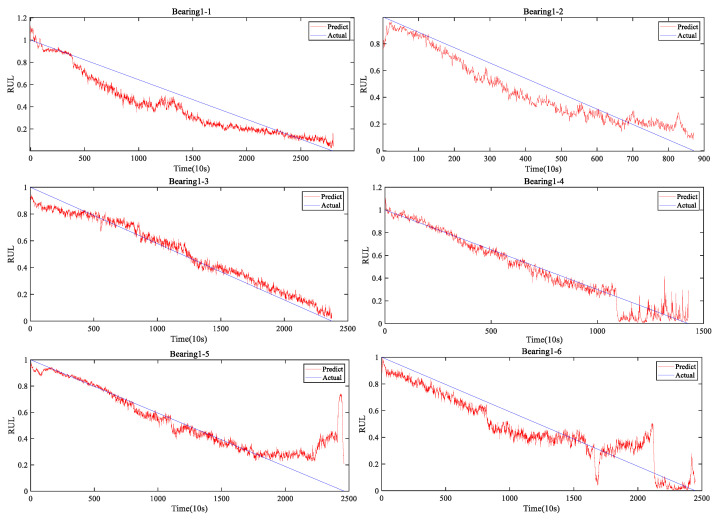

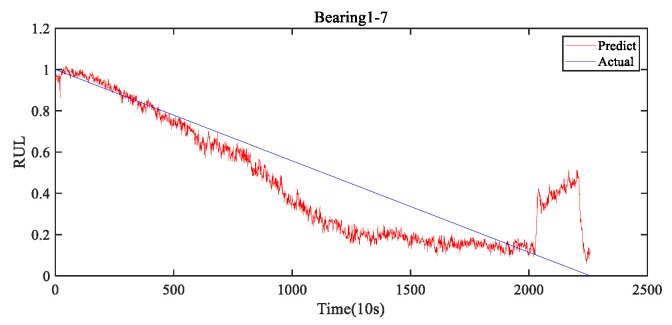
The visualization of the RUL prediction results on FRMARNet for Bearing1_1-Bearing1_7.

Figure 9 shows the visualization of the RUL prediction results for the seven bearings by the developed FRMARNet. The figure clearly shows that the proposed model accurately captured the degradation trend of the bearings. Note that the different types of bearing failures and degradation rates resulted in very different experimental durations. The FRMARNet model can capture not only long and slow degradation processes of bearings (for instance, bearing1_1 and bearing 1_6), but also rapid degradation processes (for instance, bearing1_2). Some bearings are difficult to predict when they are close to failure, which may be caused by the inconsistent pattern of bearing failure causing large differences in vibration signal fluctuations. This is followed by a quantitative comparison with other multi-scale structures, attention mechanisms, and several state-of-the-art (SOTA) RUL prediction methods.

(2) Comparison with multi-scale structures

**Figure 10:**
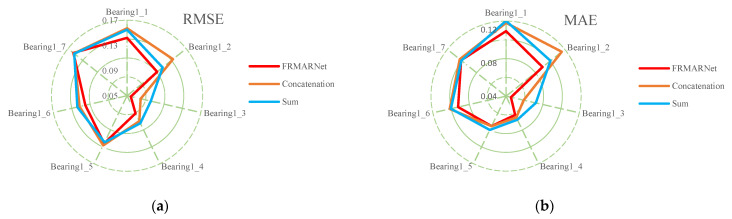
Comparison results with other multi-scale structures: (**a**) RMSE; (**b**) MAE.

**Figure 11:**
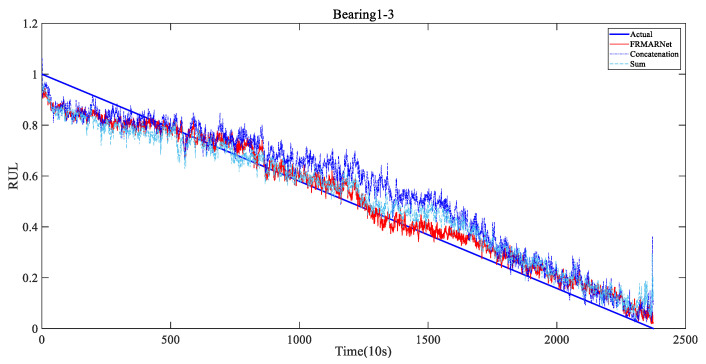
RUL prediction results of different multi-scale structures for Bearing1_3.

To verify that the FRMARNet model can improve the prediction accuracy while reducing the number of parameters, the feature reuse multi-scale attention structure in the FRMA-RBU unit is replaced by the concatenation and sum modes mentioned in Section 2.2. The rest of the network structure and hyperparameters are the same for all three methods. The average and standard deviation of the prediction results for each of the seven bearings are shown in Table 6 and Table 7 and Figure 10. Note that in FRMARNet, to enrich the feature information, the channel dimension increase ratio was 4. Due to the limitation of the computer memory capacity, the dimension increase ratio in the concatenation mode was 3, and the scale information with convolution kernels of 3, 5, and 7 was used. More scale information and a larger dimension increase ratio will significantly increase the number of parameters. As can be seen in Table 6 and Table 7, on each bearing, the RMSE and MAE of the prediction values of the proposed FRMARNet method were smaller than the other two multi-scale structures. The average RMSE decreased by 0.012 and 0.013, respectively, and the average MAE values both decreased by 0.01. In addition, the proposed method had smaller standard deviations in two evaluation metrics, which indicates that the developed model had better robustness. At the same time, the statistics of the parameter numbers show that the FRMARNet had a smaller number of parameters. The number of parameters decreased by 63.9% and 53.2% in the concatenation and sum modes, respectively, which is conducive to model optimization and reduces the computational burden. In addition, Figure 11 visualizes the RUL prediction results of different multi-scale structures for bearing1_3. Compared to the other two multi-scale structures, the FRMARNet model better captured the degradation trend of the bearings.

(3) Ablation experiments

**Figure 12:**
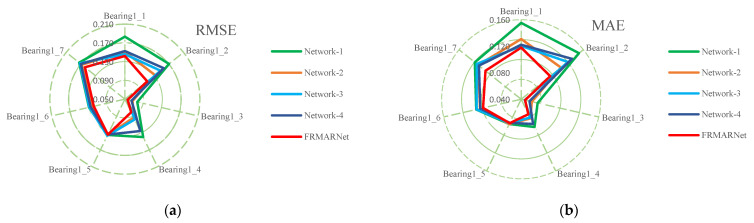
Comparison results of ablation experiments: (**a**) RMSE; (**b**) MAE.

**Figure 13:**
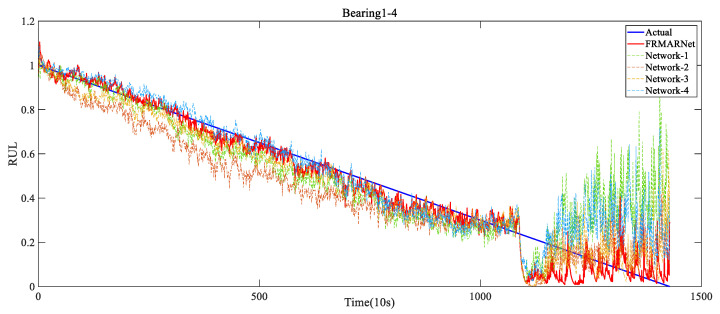
RUL prediction results of the ablation experiments for Bearing1_4.

The feature extraction capability of the model is enhanced by embedding a CMP layer and FRMA units in ResNet. Compared to the vanilla ResNet model, the developed FRMARNet model has several improvements, including the CMP layer, a feature reuse multi-scale convolution structure, and an ECA mechanism. Ablation experiments were conducted to demonstrate the effectiveness of these components in improving model prediction accuracy. We studied the impact of these differences on the prediction results by removing or changing these new improvements and keeping other parameter settings unchanged. The ablation experiments are detailed as follows:

Network-1 replaced the feature reuse multi-scale convolution structure with standard convolution and removed the ECA module. Network-2 only removed the ECA module. Network-3 only replaced the CMP layer with one-dimensional maximum pooling. Network-4 replaced the ECA module with SE attention. The detailed results of the ablation experiments are presented in Table 8 and Table 9 and Figure 12. The prediction error increased significantly when these components were removed or replaced. Specifically, comparing Network-1 with Network-2, when the feature reuse multi-scale structure was removed, the average RMSE and MAE increased by 0.02 and 0.013, respectively. The feature reuse multi-scale structure can extract different scale information from the original signal. Comparing Network-2 with the FRMARNet models, when the ECA module was removed, the average RMSE and MAE increased by 0.01 and 0.01, respectively. Comparing Network-4 with the FRMARNet models, when replacing the ECA module with SE attention, the average RMSE and MAE increased by 0.019 and 0.012, respectively. The ECA module hardly increased the parameters and was able to recalibrate scale information so that the model focused on more important scale information. Comparing Network-3 with the FRMARNet models, when the CMP layer was removed, the average RMSE and MAE increased by 0.012 and 0.009, respectively. The CMP layer was beneficial for the model to concentrate on more important feature information. The prediction results fully confirm the theoretical analysis in Section 3. In addition, Figure 13 visualizes the RUL prediction results of the ablation experiments for bearing1_4. The comparison results indicate that the FRMARNet model better captured the degradation trend of the bearings. The effectiveness of these improvements in improving the prediction accuracy of the proposed FRMARNet model can thus be demonstrated.

(4) Visualization of attention weights

**Figure 14:**
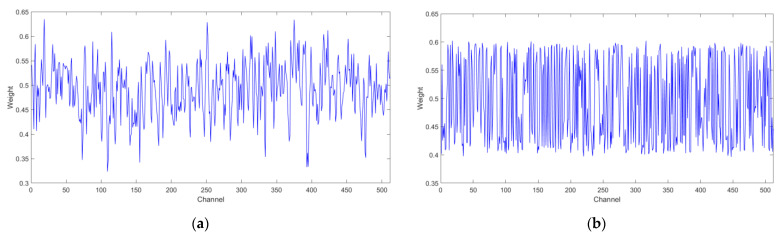
Visualization of attentional mechanisms: (**a**) bearing1_3; (**b**) bearing1_4.

The attention mechanism attempted to establish the relationship between different channels, and the attention weights reflect the focus degree on different channels. To reveal the internal operation mechanism of the attention module and prove that the model indeed learned different weights for different channels, the attention weights in the FRMA-RBU4 module are displayed visually in Figure 14, and the weights were extracted and visualized by averaging all test samples along the channel direction. Figure 14a and Figure 14b show the attention weights of bearing1_3 and bearing1_4, respectively. There was a significant difference in the weights along the channel, which indicates that the contributions of the different channels were inconsistent. The maximum value of the weight of bearing1_3 was 0.6348 for channel 19, and the minimum value was 0.3235 for channel 109. The maximum value of the weight of bearing1_4 was 0.6021 for channel 18, and the minimum value was 0.3965 for channel 447. It can be demonstrated that the ECA module has learned a group of weights with different sizes to recalibrate different channels, and adaptively endowed channels containing more degradation information with greater weights.

(5) Comparison with SOTA methods

To further demonstrate the superiority of the FRMARNet-based RUL prediction method proposed in this paper, several SOTA methods were employed for comparison, including Wu et al.’s vanilla long short-term memory (LSTM) neural networks [23], Chen et al.’s gate recurrent unit (GRU) [43], Zhu et al.’s multi-scale CNN (MSCNN) [32], Wang et al.’s deep separable ResNet (DSRN) [8], Shang et al.’s bidirectional GRU and CNN (CNN-BGRU) [42], Chen et al.’s RNN based on an encoder–decoder framework with an attention mechanism (REA) [44], Wang et al.’s feature fusion based ensemble method (FFEM) [45], and Ding et al.’s convolutional transformer (CoT) [24]. 

The results of the RUL prediction performance comparison are presented in Table 10. The comparison results show that the proposed FRMARNet model had lower average RMSE and MAE values than the other eight existing SOTA approaches, which means that the developed FRMARNet method outperformed the other bearing RUL prediction methods. Specifically, the prediction performance of the FRMARNet-based model was significantly better than that of the vanilla LSTM and GRU models, which is because the vanilla LSTM and GRU models are only able to extract long-distance time-series relationships in the data, resulting in low modeling efficiency and accuracy. For MSCNN and the multi-scale structure mentioned in Section 2.2, the prediction error was larger than that of the FRMARNet-based approach due to the complex network results, resulting in many parameters and optimization difficulties, as well as a lack of an effective mechanism to highlight important feature information. For some other compared methods, multi-scale local and global information is not extracted simultaneously, or recalibration of multi-scale information is lacking, and none of them utilize CMP or have cross-channel information interactions. This performance improvement again validates the benefits of the CMP layer, the feature reuse multi-scale structure, and the ECA module. It should be noted that, unlike some comparison methods that use signal processing to obtain artificial features, the FRMARNet model proposed in this paper uses the original signal as the input and is an end-to-end prediction framework. The methods without targeted signal processing, information entropy, and artificial feature selection lose some seemingly unimportant feature information. The raw vibration signal contains the richest information on degraded features.

## 5. Conclusions

Accurate rolling bearing RUL prediction is highly dependent on significant multi-scale feature information extracted from the original signal. The authors of this paper developed a novel lightweight FRMARNet architecture for rolling bearing RUL prediction that combines the advantages of cross-channel interaction, multi-scale features, and attention mechanisms. The CMP based on cross-channel interaction can automatically select important feature information, and the FRMA unit based on multi-scale and attention mechanisms can extract multi-scale information and recalibrate multi-scale information to emphasize important feature information and suppress redundant information. In addition, extensive experiments were carried out on the bearing dataset to validate the developed method. Comparative tests with the existing concatenation and sum multi-scale structure indicate that the average RMSE decreased by 9.5% and 10.2%, respectively, and the average MAE both decreased by 10.2%. The number of parameters decreased by 63.9% and 53.2%, respectively. The experimental results demonstrate that the proposed FRMA unit significantly improved the prediction accuracy while reducing the number of model parameters. The ablation experiments indicate that the average RMSE increased by 13.9%, 8%, and 9.5% (the MAE increased by 11.7%, 10.2%, and 9.3%) when the feature reuse multi-scale structure, ECA module, and CMP layer were removed, respectively. The effectiveness of the CMP layer, the feature reuse multi-scale structure, and the ECA module are demonstrated. The attention weight visualization shows that the model learned a set of weights of different sizes to recalibrate the scale information. Compared to the SOTA method, the average RMSE was reduced by at least 8% and the MAE was reduced by at least 5%. These results prove the superiority of the method. In summary, extensive experiments were conducted to verify the advancement and effectiveness of the developed method, as well as the necessity and reasonableness of the improvements. In future work, we need to investigate not only the effects of multi-scale information on prediction performance but also the effects of the temporal characteristics of raw data on prediction performance. The self-attention mechanism can establish long-distance dependence on the data and capture the temporal characteristics in the data. The combination of the developed method in this paper and self-attention mechanism is a promising research direction.

## Figures and Tables

**Table 1:** The characteristics of the tested bearings.

Characteristic	Numerical Value
Outside race diameter	32 mm
Inside diameter	20 mm
Thickness	7 mm
Load ratings static	2470 N
Load ratings dynamic	4000 N
Maximum speed	13,000 rpm

**Table 2:** Description of the degradation experiment dataset.

Dataset	Data Size	Accelerated Degradation Lifetime
Bearing1_1	2803 × 2560	28,030 s
Bearing1_2	871 × 2560	8710 s
Bearing1_3	2375 × 2560	23,750 s
Bearing1_4	1428 × 2560	14,280 s
Bearing1_5	2463 × 2560	24,630 s
Bearing1_6	2448 × 2560	24,480 s
Bearing1_7	2259 × 2560	22,590 s

**Table 3:** The model structure of FRMARNet.

Layer Name	Model Parameter	Input Feature Size	Output Feature Size
Conv+BN+ReLU	C(16 × 64 × 2)	(2560 × 1)	(1252 × 16)
CMP	P(3 × 2)	(1252 × 16)	(626 × 8)
FRMA-RBU1	FR(8 − L × 2 × s − 64)	(626 × 8)	(313 × 64)
FRMA-RBU2	FR(64 − 2L × 2 × s − 128)	(313 × 64)	(157 × 128)
FRMA-RBU3	FR(128 − 4L × 2 × s − 256)	(157 × 128)	(79 × 256)
FRMA-RBU4	FR(256 − 8L × 2 × s − 512)	(79 × 256)	(40 × 512)
GAP	G(40 × 1)	(40 × 512)	(1 × 512)
FC	FC(1)	(1 × 512)	(1)

**Table 4:** RMSE results of different hyperparameters.

	L = 4, s = 4	L = 4, s = 3	L = 4, s = 5	L = 3, s = 4	L = 5, s = 4
Bearing1_1	**0.142 ± 0.021**	0.144 ± 0.011	0.155 ± 0.027	0.155 ± 0.021	0.152 ± 0.009
Bearing1_2	**0.111 ± 0.019**	0.133 ± 0.018	0.143 ± 0.010	0.142 ± 0.012	0.162 ± 0.012
Bearing1_3	**0.056 ± 0.003**	0.063 ± 0.005	0.066 ± 0.007	0.065 ± 0.006	0.067 ± 0.004
Bearing1_4	**0.081 ± 0.008**	0.117 ± 0.041	0.099 ± 0.024	0.090 ± 0.015	0.086 ± 0.007
Bearing1_5	**0.134 ± 0.003**	0.139 ± 0.009	0.140 ± 0.007	0.139 ± 0.009	0.137 ± 0.008
Bearing1_6	0.118 ± 0.004	**0.115 ± 0.005**	0.125 ± 0.008	0.124 ± 0.010	0.131 ± 0.009
Bearing1_7	**0.159 ± 0.008**	0.168 ± 0.010	0.177 ± 0.013	0.165 ± 0.012	0.175 ± 0.012
Average	**0.114 ± 0.009**	0.126 ± 0.014	0.129 ± 0.014	0.126 ± 0.012	0.130 ± 0.009

**Table 5:** MAE results of different hyperparameters.

	L = 4, s = 4	L = 4, s = 3	L = 4, s = 5	L = 3, s = 4	L = 5, s = 4
Bearing1_1	**0.118 ± 0.019**	0.118 ± 0.010	0.130 ± 0.025	0.130 ± 0.018	0.127 ± 0.008
Bearing1_2	**0.096 ± 0.018**	0.118 ± 0.016	0.127 ± 0.009	0.123 ± 0.012	0.144 ± 0.010
Bearing1_3	**0.046 ± 0.003**	0.053 ± 0.006	0.054 ± 0.008	0.056 ± 0.006	0.056 ± 0.003
Bearing1_4	**0.065 ± 0.011**	0.074 ± 0.017	0.072 ± 0.013	0.066 ± 0.014	0.071 ± 0.007
Bearing1_5	**0.080 ± 0.005**	0.084 ± 0.007	0.081 ± 0.003	0.085 ± 0.006	0.082 ± 0.010
Bearing1_6	0.099 ± 0.004	**0.097 ± 0.006**	0.108 ± 0.007	0.104 ± 0.013	0.113 ± 0.011
Bearing1_7	**0.109 ± 0.008**	0.122 ± 0.009	0.134 ± 0.017	0.115 ± 0.015	0.126 ± 0.012
Average	**0.088 ± 0.010**	0.095 ± 0.010	0.101 ± 0.012	0.097 ± 0.012	0.103 ± 0.009

**Table 6:** RMSE results of different multi-scale structures.

	FRMARNet	Concatenation	Sum
Params	**308.6 k**	855.5 k	659.2 k
Bearing1_1	**0.142 ± 0.021**	0.155 ± 0.016	0.157 ± 0.020
Bearing1_2	**0.111 ± 0.019**	0.122 ± 0.016	0.143 ± 0.032
Bearing1_3	**0.056 ± 0.003**	0.089 ± 0.011	0.072 ± 0.012
Bearing1_4	**0.081 ± 0.008**	0.098 ± 0.022	0.095 ± 0.021
Bearing1_5	**0.134 ± 0.003**	0.132 ± 0.005	0.138 ± 0.007
Bearing1_6	**0.118 ± 0.004**	0.131 ± 0.008	0.127 ± 0.008
Bearing1_7	**0.159 ± 0.008**	0.157 ± 0.006	0.158 ± 0.007
Average	**0.114 ± 0.009**	0.126 ± 0.012	0.127 ± 0.015

**Table 7:** MAE results of different multi-scale structures.

	FRMARNet	Concatenation	Sum
Bearing1_1	**0.118 ± 0.019**	0.130 ± 0.015	0.128 ± 0.016
Bearing1_2	**0.096 ± 0.018**	0.108 ± 0.015	0.125± 0.029
Bearing1_3	**0.046 ± 0.003**	0.077 ± 0.011	0.062 ± 0.013
Bearing1_4	**0.065 ± 0.011**	0.071 ± 0.015	0.068 ± 0.018
Bearing1_5	**0.080 ± 0.005**	0.085 ± 0.006	0.080 ± 0.003
Bearing1_6	**0.099 ± 0.004**	0.108 ± 0.008	0.109 ± 0.008
Bearing1_7	**0.109 ± 0.008**	0.109 ± 0.010	0.111 ± 0.009
Average	**0.088 ± 0.010**	0.098 ± 0.011	0.098 ± 0.014

**Table 8:** RMSE results of ablation experiments.

	Network-1	Network-2	Network-3	Network-4	FRMARNet
Bearing1_1	0.183 ± 0.011	0.151 ± 0.016	0.146 ± 0.015	0.153 ± 0.016	**0.142 ± 0.021**
Bearing1_2	0.171 ± 0.018	0.132 ± 0.034	0.147 ± 0.014	0.156 ± 0.022	**0.111 ± 0.019**
Bearing1_3	0.077 ± 0.017	0.067 ± 0.010	0.056 ± 0.008	0.065 ± 0.013	**0.056 ± 0.003**
Bearing1_4	0.140 ± 0.035	0.094 ± 0.017	0.098 ± 0.020	0.126 ± 0.017	**0.081 ± 0.008**
Bearing1_5	0.135 ± 0.005	0.135 ± 0.002	0.137 ± 0.008	0.134 ± 0.004	**0.134 ± 0.003**
Bearing1_6	0.129 ± 0.009	0.122 ± 0.015	0.128 ± 0.008	0.125 ± 0.021	**0.118 ± 0.004**
Bearing1_7	0.175 ± 0.010	0.168 ± 0.014	0.173 ± 0.008	0.170 ± 0.005	**0.159 ± 0.008**
Average	0.144 ± 0.015	0.124 ± 0.015	0.126 ± 0.011	0.133 ± 0.014	**0.114 ± 0.009**

**Table 9:** MAE results of ablation experiments.

	Network-1	Network-2	Network-3	Network-4	FRMARNet
Bearing1_1	0.155 ± 0.006	0.131 ± 0.017	0.120 ± 0.014	0.122 ± 0.013	**0.118 ± 0.019**
Bearing1_2	0.152 ± 0.017	0.117 ± 0.031	0.130 ± 0.012	0.138 ± 0.019	**0.096 ± 0.018**
Bearing1_3	0.065 ± 0.016	0.056 ± 0.011	0.046 ± 0.009	0.053 ± 0.013	**0.046 ± 0.003**
Bearing1_4	0.086 ± 0.014	0.072 ± 0.021	0.071 ± 0.014	0.081 ± 0.017	**0.065 ± 0.011**
Bearing1_5	0.082 ± 0.006	0.081 ± 0.004	0.080 ± 0.004	0.079 ± 0.004	**0.080 ± 0.005**
Bearing1_6	0.110 ± 0.010	0.104 ± 0.016	0.109 ± 0.009	0.103 ± 0.024	**0.099 ± 0.004**
Bearing1_7	0.130 ± 0.013	0.123 ± 0.014	0.126 ± 0.010	0.122 ± 0.006	**0.109 ± 0.008**
Average	0.111 ± 0.012	0.098 ± 0.016	0.097 ± 0.010	0.100 ± 0.014	**0.088 ± 0.010**

**Table 10:** Comparison results with SOTA methods.

Methods	Average RMSE	Average MAE
Wu’s LSTM [23]	0.301	0.254
Chen’s GRU [43]	0.266	0.205
Zhu’s MSCNN [32]	0.199	0.154
Wang’s DSRN [8]	0.148	0.128
Shang’s CNN-BGRU [42]	0.131	-
Chen’s REA [44]	0.129	0.094
Wang’s FFEM [45]	0.123	0.092
Ding’s CoT(base) [24]	0.125	0.093
Proposed FRMARNet	**0.114**	**0.088**

## Data Availability

The data presented in this study are available on request from the corresponding authors.

## Abbreviations

The following abbreviations are used in this paper.

RUL	Remaining useful life
ML	Machine learning
DL	Deep learning
ResNets	Residual networks
CNNs	Convolutional neural networks
RNNs	Recurrent neural networks
FRMARNet	Feature reuse multi-scale attention residual network
FRMA	Feature reuse multi-scale attention
SGD	Stochastic gradient descent
RBU	Residual building unit
Conv	Convolution
BN	Batch normalization
ReLU	Rectified linear unit
GAP	Global average pooling
VCNN	Vanilla CNN
SMCNN	Sum multi-scale CNN
CMCNN	Concatenation multi-scale CNN
CMP	Cross-channel maximum pooling
ECA	Efficient channel attention
SE	Squeeze-and-excitation
RMSE	Root mean square error
MAE	Mean absolute error
SOTA	State-of-the-art
LSTM	Long short-term memory
GRU	Gate recurrent unit
MSCNN	Multi-scale CNN
DSRN	Deep separable ResNet
CNN-BGRU	Bidirectional GRU and CNN
REA	RNN based on an encoder–decoder framework with an attention mechanism
FFEM	Feature fusion-based ensemble method
CoT	Convolutional transformer
